# OA09 Challenging case of psoriatic arthritis: what should we do next?

**DOI:** 10.1093/rap/rkac066.009

**Published:** 2022-09-28

**Authors:** Marwa Mohareb, Anupama Nandagudi

**Affiliations:** Basildon and Thurrock Hospital, Basildon, United Kingdom; Basildon and Thurrock Hospital, Basildon, United Kingdom

## Abstract

**Introduction/Background:**

Psoriatic arthritis (PsA) is a chronic inflammatory enthesoarthro-osteopathy associated with extra-articular manifestations and several co-morbidities occurring in up to 42% of patients with psoriasis. The heterogeneity of PsA is the reason why the term ‘Psoriatic Disease’ (PsoD) has been proposed to reveal more precisely how the PsA patients suffer.

**Description/Method:**

We report a case of refractory PsA presenting with a flare raising therapeutic challenges.

In 2019, a 27-year-old man who was diagnosed since 2014 with PsA and psoriasis presented with flare up of psoriasis affecting almost all of his body and peripheral joints mainly MCPs. There was no uveitis or IBD history.

PsArc score: Tender joint count (TJC)=5, swollen joint count (SJC)=4 and VAS= 4/5

He was on methotrexate 10 mg orally once weekly that was increased to 15 mg with folic acid once weekly. Unremarkable other medical history.

Drug history included: Phototherapy without response (2007), then cyclosporine 100mg BD for six weeks which stopped due to severe mood changes.

Unsuccessful trials of 20 mg oral methotrexate once weekly because of elevated liver enzymes, subcutaneous form causing significant neurapraxia, adalimumab (2010-2013) stopped due to secondary failure, ustekinumab (2013-2014) stopped after three injections (primary failure), Infliximab (2014-2015) stopped due to throat swelling during transfusion, apremilast 30mg BD stopped due to complete inefficacy. Secukinumab was started by another trust for one year that helped his condition but he lost follow up, we arranged secukinumab continuation that was very effective for only six months and the patient presented severe flare of his skin in the form of widespread different sized erythematous indurated scaly plaques affecting all his body with PsArc score TJC=10 SJC=6, VAS was 5/5. PASI score 14.

In 2020 ixecuzimaub was started by the dermatologists that show dramatic improvement in his skin, PASI score became 1.6 but unfortunately not working for joints with 12 tender joints and evidence of tenosynovitis in almost all small joints of hands. He tried leflunomide 20 mg orally daily but stopped due to deranged liver functions.

Investigations showed fluctuating CRP levels (3-87 mg/l), normal blood counts and negative RF.

**Discussion/Results:**

We present a case of PsA that was treated by almost all synthetic and biologic DMARDS that show high efficacy in most of cases of PsA patients.  Certolizumab or golimumab were suggested by Guys and St Thomas hospital to start on. Dual biologic therapy is recently reported in the psoriatic literature including ustekinumab plus etanercept, secukinumab plus etanercept, and apremilast with all biologic agents in patients with plaque psoriasis or psoriatic arthritis not responding adequately to single biologics alone. But there was an increased incidence of urinary tract and upper respiratory infections, including a hospitalization for H2N1 flu in the ustekinumab plus etanercept case.

**Key learning points/Conclusion:**

Most physicians are often reluctant to prescribe dual biologic therapy because of safety concerns. Very little data exist regarding the safety of dual biologic medications for concomitant psoriasis and psoriatic arthritis. Additionally, the possibility of a major adverse cardiovascular event related to combined biologic therapy has been reported. More research is warranted to explore dual therapy in refractory patients.

